# Severity‐Stratified Healing Outcomes of Nurse‐Managed Negative Pressure Wound Therapy in Diabetic Foot Ulcers: A Prospective Comparative Cohort Study

**DOI:** 10.1155/nrp/8864223

**Published:** 2026-10-12

**Authors:** Suriadi Jais, Kharisma Pratama, Ivan Sujana, Suhaimi Fauzan

**Affiliations:** ^1^ Post Graduate Nursing Program Institute of Technology and Health Muhammadiyah Kalimantan Barat Pontianak Indonesia; ^2^ Faculty of Engineering Tanjungpura University Pontianak Indonesia untan.ac.id; ^3^ Faculty of Medicine School of Nursing Tanjungpura University Pontianak Indonesia untan.ac.id

**Keywords:** diabetic foot ulcer, negative pressure wound therapy, nursing practice, SHID classification, wound healing

## Abstract

**Aim:**

To compare healing outcomes of nurse‐managed negative pressure wound therapy (NPWT) and standard wound care (SWC) across baseline wound severity levels classified using the Suriadi, Haryanto, Imran Dan Defa (SHID) system in patients with diabetic foot ulcers (DFUs).

**Design:**

A prospective quasiexperimental comparative cohort study.

**Methods:**

This study was conducted at Kitamura Clinic between June and December 2025. Sixty‐three patients with DFUs were enrolled in this study (NPWT, *n* = 31; SWC, *n* = 32). Wound severity was assessed using the SHID classification system, and wound progression was monitored weekly using the depth, maceration, infection, size, tissue type, and tunnelling. The primary outcome was time to complete wound healing within 12 weeks. Kaplan–Meier survival and Cox proportional hazards regression analyses were performed. A multivariable Cox model stratified by SHID stage accounted for baseline wound severity.

**Results:**

Median healing time was significantly shorter in the NPWT group than in the SWC group (31 [13–70] vs. 81.5 [31–127] days, *p* < 0.001). All patients in the NPWT group achieved complete healing within 12 weeks compared with 50% in the SWC group (*p* < 0.001). Survival analysis demonstrated significantly faster healing in the NPWT group (log‐rank test, *p* < 0.001). In the stratified multivariable Cox model adjusting for age, hemoglobin A1c, and sex, NPWT therapy remained independently associated with accelerated wound healing (adjusted hazard ratio 2.77, 95% confidence interval 1.40–5.47; *p* = 0.003).

**Conclusion:**

Nurse‐managed NPWT was associated with faster wound healing across SHID Stages 3–5. SHID‐based severity assessment may provide useful prognostic information for interpreting healing trajectories, treatment planning, and follow‐up monitoring.

## 1. Introduction

Diabetic foot ulcers (DFUs) are among the most serious and costly complications of diabetes mellitus (DM) and remain a major public health concern worldwide. DFUs are associated with delayed healing, recurrent infection, increased risk of lower‐extremity amputation, and premature mortality [1]. The pathophysiology of DFUs is multifactorial, involving peripheral neuropathy, vascular impairment, dysregulated inflammatory responses, and altered cytokine and growth factor activity. These mechanisms collectively contribute to delayed wound healing and chronic wound environments [2–4]. Consequently, DFUs substantially reduce patients’ quality of life and impose a considerable burden on healthcare systems [5, 6].

Negative pressure wound therapy (NPWT), most commonly delivered through vacuum‐assisted closure (VAC) systems, is an established adjunctive modality for DFU management. NPWT promotes wound healing by improving local perfusion, reducing oedema, removing exudates, decreasing bacterial load, and stimulating granulation tissue formation [7–12]. Compared with conventional wound care, NPWT accelerates wound closure and may reduce the incidence of major amputation [9]. These advances reflect continuing efforts to refine wound care technologies and improve clinical outcomes in patients with complex diabetic wounds [13].

In clinical nursing practice, wound assessment and ongoing monitoring are central to DFU management. Nurses play a critical role in evaluating wound severity, selecting appropriate adjunctive therapies, such as NPWT, and monitoring treatment response over time. Therefore, understanding how baseline wound severity influences healing trajectories under nurse‐managed NPWT is essential for supporting evidence‐based wound care nursing practice.

Accurate wound severity assessment is essential for selecting appropriate treatment strategies and predicting healing outcomes in DFU management [14, 15]. The Wagner classification system has been widely used to grade DFUs according to wound depth and the presence of infection or gangrene [14, 16]. However, reported limitations in inter‐observer reliability and prognostic accuracy, particularly for healing duration and treatment response, restrict its clinical utility [14, 17]. These limitations highlight the need for classification systems that are reliable, clinically informative, and useful for predicting wound outcomes.

The Suriadi, Haryanto, Imran, and Defa (SHID) diabetic wound classification system was developed to address these limitations. Compared with commonly used systems, including the Wagner and University of Texas classifications, SHID has demonstrated superior validity and reliability in patients with DFUs [14]. Among nurses, the SHID classification showed almost perfect inter‐rater agreement (Kappa = 0.81; 95% confidence interval [CI]: 0.65–0.97), exceeding that of the Wagner scale (Kappa = 0.77; 95% CI: 0.52–0.96). Furthermore, the predictive validity studies of SHID classification showed good diagnostic performance, with a sensitivity of 74% and specificity of 97% at a cut‐off score of ≤ Grade 2 [14]. By comparison, the Wagner classification at a cut‐off score of ≥ 2 showed a sensitivity of 58% and specificity of 77% [17]. Importantly, SHID‐classified wound severity correlates with clinically relevant outcomes, including healing time [17].

The SHID classification categorizes diabetic wounds into six grades according to wound depth, tissue involvement, and the presence of infection, ischemia, or osteomyelitis. Grade 1 refers to wounds limited to the epidermis and/or dermis. Grade 2 includes epidermal and/or dermal wounds accompanied by one or more signs of infection, ischemia, or osteomyelitis confirmed by X‐ray examination. Grade 3 involves deeper tissue structures, including subcutaneous tissue, fascia, muscle, or tendon. Grade 4 includes these same structures with accompanying infection, ischemia, or radiographically confirmed osteomyelitis. Grade 5 extends to deeper anatomical structures, including the joint capsule and bone. Grade 6 represents the most severe category and involves subcutaneous tissue, fascia, muscle, tendon, joint capsule, or bone, together with one or more signs of infection, ischemia, or osteomyelitis confirmed by X‐ray examination [14]. Because more severe wounds are associated with longer healing periods and greater utilization of healthcare resources, early and accurate severity assessment is important for guiding timely and appropriate intervention [15].

Previous studies have established NPWT effectiveness for DFUs and shown that wound severity influences healing outcomes [10, 18]. However, limited evidence describes NPWT outcomes across standardized baseline severity categories using SHID. Severity‐stratified healing trajectories may provide clinically relevant information for prognosis and treatment planning, particularly in nurse‐managed, real‐world wound care. Therefore, this study aimed to compare healing outcomes between nurse‐managed NPWT and standard wound care (SWC) across baseline wound severity stages classified using the SHID system.

DFU healing is further influenced by systemic factors, including glycemic control, with poor glycemic status consistently associated with delayed wound healing and adverse clinical outcomes [19, 20]. In parallel, emerging adjunctive therapies, including topical insulin, bioengineered tissues, and advanced wound monitoring technologies, continue to be explored to enhance healing in complex diabetic wounds [1, 3, 19, 20]. Preventive strategies, including thermography‐based foot care protocols, further support the need for individualized and severity‐driven DFU management [21–23].

## 2. Materials and Methods

### 2.1. Design

This prospective comparative cohort study evaluated differences in time‐to‐healing according to baseline wound severity among patients with DFUs who received nurse‐managed NPWT or SWC. The SHID classification was used to assess baseline severity and stratify patients into severity categories. Clinical outcomes were monitored until complete healing or study completion. Eligible patients were allocated to either the NPWT or SWC group. The NPWT system was implemented following consultation with wound care specialists at the Kitamura Wound Clinic, and NPWT was applied by specialized wound care nurses. Patients in the SWC group received conventional wound care according to routine clinical protocols.

### 2.2. Setting and Participants

This study was conducted at the Kitamura Wound Clinic in Pontianak City, West Kalimantan, Indonesia, between June and December 2025. Participants were recruited from the clinic using purposive sampling. Eligible patients were allocated to the intervention or control group based on the study setting and the feasibility of implementing the intervention. Baseline characteristics were compared between the groups to assess comparability before intervention implementation. A sensitivity power analysis was conducted in Stata for a two‐sample comparison of proportions with a two‐sided *p* value of 0.05 [24]. With a total sample size of 63 participants, the study achieved 80% power to detect a large absolute between‐group difference of approximately 30% points in the healing proportion. The expected effect size was based on previous studies demonstrating improved healing outcomes with NPWT in patients with DFUs, including the trial by Blume et al., which reported higher wound closure rates with NPWT than with advanced moist wound therapy (43.2% vs 28.9%) [25].

Smaller between‐group differences were associated with substantially lower statistical power. Patients were eligible if they presented to the clinic for the first time with a diabetic wound, regardless of whether the wound was recurrent or new. No age restrictions were imposed. Patients were excluded if they had vascular or ischemic disorders, defined as an ankle–brachial index (ABI) < 0.90 indicating mild to moderate peripheral arterial disease (PAD), osteomyelitis, or systemic complications such as cardiac or renal diseases.

### 2.3. Data Collection

Data were collected by two research assistants who had received specialized training in diabetic wound care. Data collection included demographic information, physical examinations, wound classification, and weekly monitoring of wound progression. Physical assessments included measurement of the ABI using vascular Doppler and assessment of neuropathy status using a 10 g monofilament test. Diabetic wounds were classified using the SHID classification system, and wound progression was monitored weekly using the depth, maceration, inflammation/infection, size, tissue type of the wound bed, type of wound edge, and tunnelling/undermining (DMIST) [26]. Hemoglobin A1c (HbA1c) levels were measured by laboratory personnel.

### 2.4. Statistical Analysis

Categorical variables were presented as frequencies and percentages. Continuous variables were presented as mean ± standard deviation (SD) or median (min–max), depending on data distribution as assessed using the Shapiro–Wilk test. Baseline differences between the groups were analyzed using the chi‐square test or Fisher’s exact test for categorical variables and the Mann–Whitney U test for continuous variables. Healing time was compared using the Mann–Whitney U test, and the proportion of patients who achieved healing within 12 weeks was compared using Fisher’s exact test. Stratified comparisons by SHID stage were performed using the Mann–Whitney U test for between‐group comparisons and the Kruskal–Wallis test for within‐group comparisons. These analyses were considered exploratory because of limited and unequal sample sizes across severity strata.

Time‐to‐healing was evaluated using Kaplan–Meier analysis and compared using the log‐rank test. Cox proportional hazards regression was performed to identify independent predictors of healing. Because the SHID stage was used as a stratification factor in the survival analysis, a stratified Cox proportional hazards model was applied to account for potential nonproportionality across wound severity strata. Hazard ratios (HRs) with 95% CIs are reported. Statistical significance was defined as a two‐sided *p* value of < 0.05. Analyses were conducted using Stata Version 17.0. Software (Stata, College Station, TX, USA).

### 2.5. Ethical Consideration

The study protocol was reviewed and approved by the Health Research Ethics Committee of the Muhammadiyah Institute of Technology and Health, West Kalimantan (Approval No. 123). All study procedures were conducted in accordance with the ethical standards and institutional guidelines.

## 3. Results

### 3.1. Characteristics of the Study Participants

A total of 63 patients with DFUs were included in this study (NPWT, *n* = 31; SWC, *n* = 32). The baseline demographic and clinical characteristics are shown in Table 1. The groups were generally comparable, with no statistically significant differences in age, sex, smoking status, systolic or diastolic blood pressure, HbA1c level, ABI, duration of diabetes, education level, religion, wound location, or wound triggers (all *p* > 0.05). However, baseline wound severity differed significantly between groups. The distribution of SHID classification differed between groups (*p* = 0.008), and the NPWT group had a higher mean DMIST score than the SWC group (6.0 ± 0.9 vs 5.0 ± 1.3; *p* = 0.003). No serious adverse events related to NPWT or SWC were observed during the study period. Specifically, no patients experienced major bleeding, secondary wound infection requiring treatment discontinuation, device‐related complications, or severe pain necessitating withdrawal from therapy. Mild discomfort during dressing changes was occasionally reported in both groups; however, these events were transient and did not interfere with treatment continuation.

**TABLE 1:** Characteristic of the participants.

Variable	NPWT group	SWC group	*p* value
Sex, *n* (%)			
Male	11 (35.50)	17 (53.10)	0.350^a^
Female	20 (64.50)	15 (46.90)	
Age (years), median (min–max)	48 (31–76)	52 (44–85)	0.127^b^
Healing time (days), median (min–max)	31 (13–70)	81.5 (31–127)	< 0.001^b^, ^∗∗^
DMIST, mean (SD)	6.0 ± 0.9	5.0 ± 1.3	0.003^c^, ^∗^
Systolic blood pressure (mmHg), median (min–max)	125 (110–140)	125 (110–140)	0.267^b^
Diastolic blood pressure (mmHg), median (min–max)	80.5 (70–80)	80.5 (70–80)	0.246^b^
HbA1c (%), median (min–max)	8.9 (8.3–10.9)	8.9 (8.3–10.9)	0.215^a^
ABI, *n* (%)			
< 0.9	0 (0.0)	0 (0.0)	0.460^a^
0.9–1.3 (Normal)	31 (100)	32 (100)	
Smoking, *n* (%)			
Yes	0 (0.0)	4 (12.50)	0.113^a^
No	31 (100)	28 (87.50)	
> 1.3	0 (0.0)	0 (0.0)	
Duration of DM, *n* (%)			
≤ 5 years	31 (100)	30 (93.8)	0.180^a^
> 5 years	0 (0.0)	2 (6.2)	
Religion, *n* (%)			
Muslim	15 (48.39)	20 (62.50)	0.222^a^
Christian	12 (38.71)	6 (18.75)	
Buddhist	4 (12.90)	6 (18.75)	
Education, *n* (%)			
Elementary school	12 (38.7)	11 (34.4)	0.150^a^
Junior high school	8 (25.8)	0 (0.0)	
Senior high school	10 (32.3)	12 (37.5)	
Higher education	1 (3.2)	9 (28.1)	
Wound location, *n* (%)			
Forefoot	9 (29.3)	13 (29.03)	0.269^a^
Midfoot	12 (38.71)	8 (25.00)	
Hindfoot	7 (22.58)	4 (13.50)	
Other location	3 (9.68)	7 (21.88)	
Trigger of wound, *n* (%)			
Trauma	31 (100)	29 (90.62)	0.238^c^
Unknown	0 (0)	3 (9.38)	
Ulcer			
New	31 (100)	32 (100)	NA
Recurrence ulcer	0 (0)	0 (0)	
SHID diabetic wound classification, *n* (%)			
Stage 3	13 (41.94)	22 (68.75)	0.008^a^
Stage 4	12 (38.71)	2 (6.25)	
Stage 5	6 (19.35)	8 (25.00)	
Time to healing (weeks)			
< 12 weeks	31 (100)	16 (50.0)	*p* < 0.001^c^, ^∗∗^
≥ 12 weeks	0 (0)	16 (50.0)	

^∗^significant *p* value < 0.05.

^∗∗^significant *p* value < 0.001.

^a^Chi Square.

^b^Mann–Whitney U.

^c^Fisher’s exact test. NA (not applicable).

### 3.2. Overall Wound Healing Outcomes

Kaplan–Meier survival curves demonstrated time to complete wound healing during the 12‐week follow‐up period. The NPWT group exhibited a markedly faster decline in the probability of remaining unhealed than the SWC group did (Figure 1). The between‐group difference was statistically significant (log‐rank *χ* ^2^ = 37.19, *p* < 0.001). Restricted mean survival time (RMST) analysis truncated at 70 days showed a significantly shorter wound healing duration in the NPWT group than in the SWC group. The RMST was 35.0 days in the NPWT group and 65.3 days in the SWC group, corresponding to a between‐group difference of 30.3 days (95% CI 22.7–37.9; *p* < 0.001).

**FIGURE 1:**
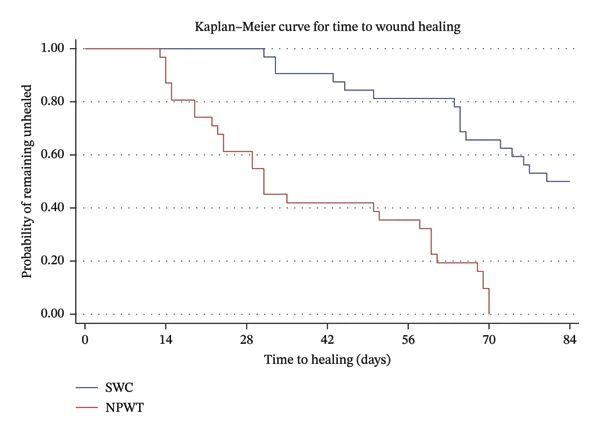
Kaplan–Meier survival curves showing time to complete wound healing during the 12‐week follow‐up period. The NPWT group demonstrated significantly faster wound healing compared with SWC (log‐rank *χ* ^2^ = 37.19, *p* < 0.001). RMST analysis truncated at 70 days demonstrated a significantly shorter wound healing duration in the NPWT group than in the SWC group.

### 3.3. Cox Proportional Hazards Model

Cox proportional hazards regression analysis demonstrated that patients receiving NPWT had significantly faster wound healing than those receiving SWC (Table 2). In the unadjusted model, patients treated with NPWT had a 6.80‐fold higher probability of wound healing over time than those in the control group (HR 6.80, 95% CI 3.35–13.79; *p* < 0.001). After adjustment for age, HbA1c level, and sex, NPWT remained independently associated with accelerated wound healing (adjusted HR 2.77, 95% CI 1.40–5.47; *p* = 0.003). Age was modestly but significantly associated with healing outcome (adjusted HR 1.02, 95% CI 1.00–1.05; *p* = 0.043), whereas HbA1c level and sex were not significantly associated with healing duration. Assessment of the proportional hazards assumption using Schoenfeld residuals demonstrated no significant violation of the proportional hazards assumption (global test *p* = 0.814).

**TABLE 2:** Cox proportional hazards model for time to wound healing.

Variable	Unadjusted HRs (95% CI)	*p* value	Adjusted HRs (95% CI)	*p* value
NPWT vs Control	6.80 (3.35–13.79)	< 0.001	2.77 (1.40–5.47)	0.003
HbA1c (per 1% increase)	—	—	1.03 (0.84–1.28)	0.756
Age (per 1‐year increase)	—	—	1.02 (1.00–1.05)	0.043
Female vs Male	—	—	1.16 (0.67–2.03)	0.592

### 3.4. Wound Healing Time According to SHID Classification

Stratified analysis according to SHID classification demonstrated consistently shorter wound healing duration in the NPWT group than in the SWC group across all SHID stages (Table 3). Among patients with Stage 3 ulcers, the median healing time was 31 (range 13–70) days in the NPWT group compared with 72 days (range 33–120) in the SWC group, with complete healing within 12 weeks achieved in all NPWT‐treated patients versus 63.6% of patients receiving SWC. Similar patterns were observed among patients with Stage 4 ulcers, for whom the median healing time was 24 days in the NPWT group and 80 days in the SWC group. Among patients with Stage 5 ulcers, the NPWT group also had a shorter healing duration and substantially higher rates of complete healing within 12 weeks than the SWC group. Censoring occurred predominantly in the SWC group, particularly among patients with Stage 5 ulcers, in whom 87.5% of wounds did not achieve complete healing within the 12‐week follow‐up period. Overall healing duration differed significantly across SHID stages (Kruskal–Wallis *χ* ^2^ = 19.27, *p* < 0.001). Post hoc pairwise comparisons demonstrated significant differences between Stage 3 and Stage 4 ulcers and between Stage 4 and Stage 5 ulcers, whereas no statistically significant difference was observed between Stage 3 and Stage 5 ulcers.

**TABLE 3:** Wound healing outcomes stratified by SHID classification.

SHID stage	NPWT (*n*)	Healing time median (min–max), days	Complete healing ≤ 12 weeks *n* (%)	Censored *n* (%)	SWC (*n*)	Healing time median (min–max), days	Complete healing ≤ 12 weeks *n* (%)	Censored *n* (%)	*p* value
Stage 3	13	31 (13–70)	13 (100)	0 (0)	22	72 (33–120)	14 (63.6)	8 (36.4)	< 0.001
Stage 4	12	24 (14–61)	12 (100)	0 (0)	2	80 (43–127)	1 (50.0)	1 (50.0)	< 0.001
Stage 5	6	69 (29–70)	6 (100)	0 (0)	8	120 (65–123)	1 (12.5)	7 (87.5)	0.002

*Note:* Overall healing duration differed significantly across SHID stages (Kruskal–Wallis *χ* ^2^ = 19.27, *p* < 0.001). Post hoc pairwise comparisons using Mann–Whitney rank‐sum tests with Bonferroni correction demonstrated significant differences between Stages 3 and 4 ulcers (*p* < 0.001) and between Stages 4 and 5 ulcers (*p* = 0.0005), whereas no statistically significant difference was observed between Stages 3 and 5 ulcers (*p* = 0.267). Censored cases represented wounds that did not achieve complete healing within 12 weeks of follow‐up. *p* values for subgroup comparisons were derived from survival analyses using log‐rank tests. These subgroup analyses were exploratory and were not adjusted for multiplicity.

### 3.5. Kaplan–Meier Analysis Stratified by SHID Classification

Additional Kaplan–Meier analysis stratified by SHID Stage 3 ulcers demonstrated a more rapid decline in the probability of remaining unhealed in the NPWT group than in the SWC group throughout the follow‐up period (Figure 2).

**FIGURE 2:**
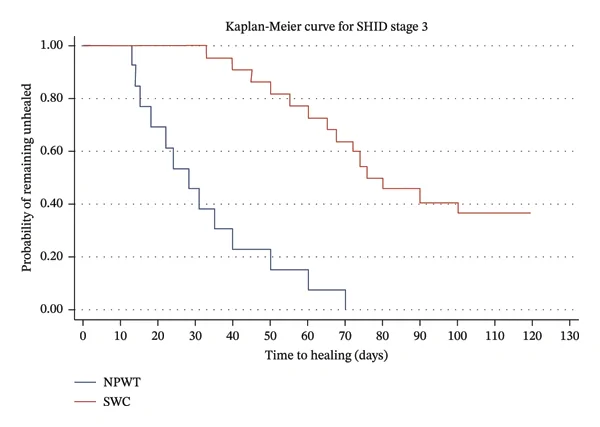
Kaplan–Meier survival curves stratified by SHID Stage 3 ulcers. Patients treated with NPWT demonstrated significantly faster wound healing than those treated with SWC (log‐rank *χ* ^2^ = 7.64, *p* = 0.006).

## 4. Discussion

This study demonstrated that NPWT was associated with substantially faster wound healing and higher rates of complete wound closure than SWC among patients with DFUs across SHID‐defined wound severity categories. Patients treated with NPWT had shorter healing times and lower censoring rates. Kaplan–Meier, Cox proportional hazards, and RMST analyses consistently showed more favorable outcomes with NPWT. These findings support previous evidence that NPWT accelerates DFU healing and extend this evidence by incorporating severity stratification using a validated wound classification system [26, 27].

Kaplan–Meier survival analysis demonstrated earlier wound closure in the NPWT group than in the SWC group throughout the 12‐week follow‐up period. Additional RMST analysis, which does not rely on the proportional hazards assumption, confirmed a shorter healing duration in the NPWT group. Consistency across these analytical approaches strengthens the robustness of the findings and suggests that the observed treatment association was not dependent on a single statistical model.

The beneficial effects of NPWT on wound healing are biologically plausible and have been consistently reported in previous studies. NPWT promotes angiogenesis, reduces oedema, decreases bacterial burden, enhances granulation tissue formation, and stimulates wound contraction, all of which are central to the repair of chronic diabetic wounds [3, 18, 27–32]. Continuous exudate removal and improved local tissue perfusion may also reduce infection risk and optimize the wound microenvironment [18, 33]. Randomized controlled trials and meta‐analyses have reported approximately 40%–50% higher healing rates and faster wound closure in DFUs treated with NPWT than in those treated with conventional dressings [3, 18, 31, 32]. The present findings are consistent with this body of evidence and suggest that these benefits may extend to patients with relatively severe ulcers.

An important strength of this study is the integration of SHID‐based wound severity classification with time‐to‐healing analysis. The SHID classification system has demonstrated superior validity and reliability compared with several conventional DFU classification systems, including the Wagner classification [14]. By stratifying healing outcomes according to SHID stages, this study provided a more clinically informative assessment of healing outcomes across different levels of baseline wound severity.

Stratified subgroup analysis demonstrated consistently shorter healing duration and higher 12‐week complete healing rates in the NPWT group across Stages 3–5 ulcers. In particular, all patients with Stage 3 ulcers who received NPWT achieved complete healing during follow‐up, whereas a substantial proportion of those receiving SWC remained unhealed. Additional Kaplan–Meier subgroup analysis stratified according to SHID stage 3 ulcers also showed faster healing in the NPWT group.

The SHID‐stratified findings provide clinically relevant insight into the relationship between baseline severity and healing. Greater SHID severity was associated with longer healing times and lower complete‐healing rates in the SWC group, whereas NPWT was associated with faster healing across Stages 3–5. These findings support the prognostic relevance of baseline wound severity and suggest that SHID may provide a structured framework for characterizing severity, anticipating healing trajectories, and guiding treatment planning and follow‐up.

These severity‐stratified findings should be interpreted cautiously because subgroup sizes were small and unequal. Notably, Stage 4 ulcers had a shorter median healing time than Stage 3 ulcers in the NPWT group, despite greater severity generally being associated with prolonged healing. Several factors may explain this finding. First, the Stage 4 subgroup included only two patients in the SWC group, creating substantial imbalance and limiting statistical stability. Second, DFU healing depends not only on structural severity but also on tissue perfusion, infection burden, wound location and size, vascular status, and individual response to NPWT [34, 35]. These factors may influence healing independently of SHID stage. Therefore, the shorter healing time observed for Stage 4 ulcers should be considered exploratory rather than evidence of a linear relationship between SHID severity and healing time.

The present study also incorporated baseline DMIST scores to provide additional information on wound bed complexity and tissue condition at study entry. Patients in the NPWT group demonstrated higher baseline DMIST scores, suggesting greater inflammatory burden, tissue compromise, and exudate severity at baseline. Despite this greater wound complexity, the NPWT group achieved superior healing outcomes. However, because longitudinal DMIST monitoring data were not systematically analyzed, this study could not determine whether changes in DMIST scores mediated or reflected treatment responsiveness over time. Mechanistic interpretations regarding dynamic changes in wound severity should therefore remain cautious.

In the adjusted Cox proportional hazards model, NPWT remained independently associated with accelerated wound healing after adjustment for age, sex, and HbA1c level. This finding indicates that the association between NPWT and faster healing persisted after accounting for key patient‐level covariates. Together with the severity‐stratified analyses, this result supports an independent association between NPWT and healing, although causal interpretation remains limited by the quasiexperimental design. Age was also independently associated with wound healing outcome, although the magnitude of the effect was modest. In contrast, HbA1c level and sex were not significant predictors in the adjusted model.

Although the overall Cox model demonstrated a significant treatment effect, the small and imbalanced SHID subgroups limited assessment of whether treatment effects differed by severity. Accordingly, subgroup‐specific effects should be interpreted cautiously. RMST analysis was included to address this limitation because it does not depend on the proportional hazards assumption. The consistency of the RMST findings with the primary survival analyses further supports the beneficial association between NPWT and wound healing.

Subgroup‐specific adjusted HR analyses and forest plot analyses were not performed because the small strata would have produced unstable estimates and increased the risk of overinterpretation. Therefore, the subgroup findings should be interpreted primarily as exploratory observations rather than confirmatory evidence of differential treatment effects. Larger stratified cohorts are needed to determine whether the effectiveness of NPWT varies by DFU severity stage and to clarify potential heterogeneity of treatment effects across wound severity strata.

Despite multivariable adjustment and stratified modeling, residual confounding cannot be excluded because of the quasiexperimental design. Nutritional status, microvascular perfusion, infection burden, and adherence to off‐loading protocols were not comprehensively assessed and may have influenced healing. However, the consistent treatment effect after severity stratification and covariate adjustment supports the robustness of the observed association, although residual confounding remains possible.

The findings have important implications for nursing and wound care practice. Integrating structured wound assessment, including SHID classification and DMIST evaluation, may support severity‐based DFU management. They also support the role of trained wound care nurses in implementing and monitoring NPWT to promote healing and improve outcome prediction. Baseline SHID assessment may facilitate prognostic communication, treatment planning, healing monitoring, patient counseling, and identification of patients requiring treatment escalation or multidisciplinary care. However, NPWT should not be applied to all DFUs regardless of clinical characteristics; treatment selection should be guided by comprehensive wound assessment and clinical judgment.

This study has several strengths. First, integrating a validated wound severity classification with time‐to‐healing analysis enabled severity‐stratified evaluation of treatment outcomes. Second, the use of Kaplan–Meier analysis, Cox proportional hazards regression, and RMST analysis strengthened the assessment of time‐to‐event outcomes and provided complementary estimates of treatment effect. Third, subgroup stratification according to SHID severity stages provided clinically relevant insight into the performance of NPWT across varying levels of wound severity. Finally, the real‐world clinical setting enhances the relevance of the findings for routine DFU care.

However, several limitations should be acknowledged. The quasiexperimental design without randomization may have introduced residual confounding despite baseline comparability between groups. Treatment allocation was determined by clinical implementation feasibility rather than random assignment. The study was conducted at a single centre with a relatively modest sample size, particularly within individual SHID strata, which limited subgroup‐specific analyses and reduced statistical stability. Small and unequal subgroup sizes also prevented formal assessment of whether SHID severity modified the NPWT treatment effect. Longitudinal DMIST monitoring data were not systematically analyzed, limiting interpretation of dynamic wound progression and treatment response. Long‐term outcomes, including ulcer recurrence, amputation rates, and cost‐effectiveness, were also not evaluated. Future multicentre randomized studies with larger sample sizes, longitudinal wound monitoring, and extended follow‐up are warranted to confirm these findings and further evaluate the long‐term clinical and economic implications of NPWT in DFU management.

## 5. Conclusion

NPWT was associated with significantly faster wound healing and higher proportions of complete wound closure than SWC among patients with DFU across SHID severity stages. Kaplan–Meier analysis, Cox proportional hazards regression, and RMST analysis consistently demonstrated superior healing outcomes in the NPWT group than in the SWC group. Integrating SHID‐based wound severity classification with time‐to‐healing analysis provided clinically meaningful evidence on severity‐stratified treatment outcomes. However, these findings should be interpreted cautiously because of subgroup imbalance and limited sample sizes within several SHID strata.

The findings support combining structured baseline wound severity assessment with advanced wound care to inform prognosis, treatment planning, and monitoring in DFU management. Future multicentre randomized studies with larger stratified cohorts and longitudinal wound monitoring are warranted to confirm these findings and clarify whether NPWT treatment effects differ across severity stages.

## Author Contributions

All authors made substantial contributions to the conception and design, acquisition of data, analysis and interpretation of data, drafting of the manuscript, and critical revision of important intellectual content.

## Funding

This study was supported by the Ministry of Higher Education, Science, and Technology (Grant No.: 0070/C3/AL.04/2025).

## Disclosure

All authors have read and approved the final version of the manuscript.

## Ethics Statement

Ethics approval was obtained from the Local Ethics Committee (no 123).

## Consent

All participants provided written informed consent before participating in the interviews in accordance with the Declaration of Helsinki and institutional ethical guidelines.

## Conflicts of Interest

The authors declare no conflicts of interest.

## Data Availability

The data supporting the findings of this study are available from the corresponding author upon request.
